# Evaluation of image lag in a flat‐panel, detector‐equipped cardiovascular X‐ray machine using a newly developed dynamic phantom

**DOI:** 10.1120/jacmp.v16i2.5213

**Published:** 2015-03-08

**Authors:** Hisaya Sato, Keisuke Kondo, Kyoichi Kato, Yasuo Nakazawa

**Affiliations:** ^1^ Graduate School of Medical Health Sciences, Komazawa University Setagaya‐Ku Tokyo Japan; ^2^ Graduate School of Nursing and Rehabilitation Sciences, Showa University Shinagawa‐ku Tokyo Japan

**Keywords:** image quality, noise, fluoroscopy, angiography

## Abstract

We developed a dynamic phantom for use in routine checks. This phantom can be used to physically evaluate image lag that occurs in dynamic images. It has a unique measurement method. ROIs on the target are chosen, and, with the position of ROIs fixed on the image, changes in pixel value are detected physically when the target passes through the ROIs over time and perceived as image lag. Thus, it was possible to physically detect different intensities of image lag lasting less than one second while maintaining the same intensity trends. The checking technique we propose with the dynamic phantom that we developed could be effective for routine checking of fluoroscopy X‐ray machines, and could become an established method.

PACS number: 87.59.C‐, 87.59.Dj

## I. INTRODUCTION

Flat‐panel detector (FPD)‐equipped cardiovascular X‐ray machines (hereinafter, “fluoroscopy X‐ray machines”) are continually becoming more advanced as coronary artery treatment techniques improve. The self‐check function that fluoroscopy X‐ray machines automatically perform is used in place of checks before use (hereinafter, “routine checks”).

Image lag is an important factor in dynamic fluoroscopy, but currently there are no guidelines for routinely checking. Regardless of conversion method and their capabilities, lag phenomenon is known to degrade the FDP fluoroscopic images. Cause of lag has been previously described by Zhao et al.^1^, ^2^, ^3^, ^4^, ^5^ and others. There are two types of lag phenomenon: the instantaneous lag and the long‐term lag. Instantaneous lag occurs within seconds to minutes and disappear instantaneously and thus are extremely hard to capture and evaluate quantitatively. Long‐term lag occurs by accumulation of X‐ray information to the FDP over hours, months, and years and is thus much easier to document. Instantaneous lag can occur within the FDP, in the circuit, and during image processing. For instance, in the direct conversion method, residual target within the panel may be superimposed on the subsequent X‐ray exposure causing instantaneous lag. In the indirect conversion method, light generated within CSI may linger till next exposure. Lags within the circuit are processed immediately and will be incorporated into the image. Instantaneous lag during image processing happens by creation of ghost images, as in recursive filter.

More recent machines utilized variable parameters and thus have multiple factors contributing to creation of instantaneous lag.

In cardiac catheter setting, instantaneous lag may result in image blur and even pseudolesions adjacent to a vessel, mimicking aneurysms or dissection, and can have serious clinical implications.

Therefore, routine checking and remedy of instantaneous lag is preferable but, to date, there are no phantoms that can quantitatively asses the magnitude of instantaneous lag in dynamic fluoroscopic images. In order to quantitatively asses instantaneous lag, a phantom^6^, ^7^, ^8^ with motions ranging within seconds to minutes is required.

In this study, we have devised a dynamic phantom to quantitatively asses instantaneous lag and evaluated its feasibility in clinical application. From the results we obtained, we hope to further accumulate data and set up a reference standard for routine check setting in the future.

## II. MATERIALS AND METHODS

### A. Fluoroscopy X‐ray machine and control

The fluoroscopy X‐ray machine used was a Trinias C12 by Shimadzu (Tokyo, Japan). The types of X‐ray power control used for the fluoroscopy X‐ray machine were continuous control and pulse control which is the main type of power control used today. Pulse control is a control method in which exposure occurs from several times to several tens of times a second. Fluoroscopic and plain radiographic images are visualized as dynamic images by continually displaying single images obtained with pulse control.

### B. Image collection methods for X‐ray fluoroscopy

**Figure 1:**
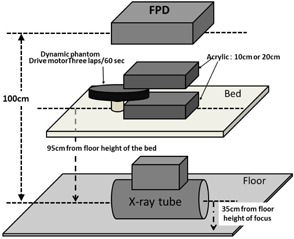
Geometry for collecting fluoroscopic X‐ray images.

The geometry for collecting fluoroscopic X‐ray images is shown in Fig. 1. The dynamic phantom was constant at the height of the isocenter.

Two 5 cm and 10 cm thick Acrylic phantoms were placed on top and the bottom of the dynamic phantom (total of 10 and 20 cm acrylic phantoms, respectively) to account for the effect of scattered rays in the human body. The dynamic phantom was put in motion between two acrylic phantoms.

The fluoroscopic X‐ray parameters used in clinical coronary artery treatments were applied, and fluoroscopic images were obtained at a pulse rate of 15 frames per second. X‐ray fluoroscopy was performed on the moving dynamic phantom for 20 s and 300 image frames were obtained. An 8 inch field of view was used. The X‐ray dose used was X‐ray entrance dose of 24.6 C/kg min on the anterior surface of the FPD, not including back scattered radiation, at a focus‐to‐FPD distance of 100 cm. The fluoroscopy parameters displayed on the fluoroscopy X‐ray machine in this experiment were 80 kV and 5 mA for phantom thickness of 20 cm, and 72 kV and 4.2 mA ms for phantom thickness of 10 cm. Fluoroscopic X‐ray images were collected using a function of the machine that could save images for 20 s. Each pulse image (300 frames, 20 s) of the collected fluoroscopic X‐ray images was saved as 1024×1024 pixel, 10‐bit uncompressed image in digital imaging and communication in medicine (DICOM) format.

X‐ray fluoroscopic images are log linear and linear portion was used for “effective exposure conversion”.

### C. Dynamic phantom developed for this study

**Figure 2:**
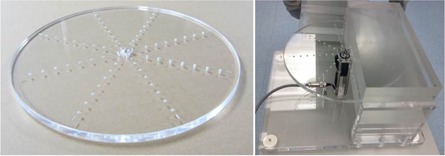
Outer appearance of the dynamic phantom that was created.

**Figure 3:**
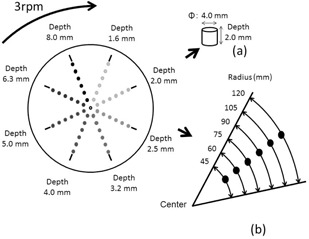
Structure of the dynamic phantom (a); function of the dynamic phantom (b).

Figure 2 shows the outer appearance of the dynamic phantom. Figure 3(a) shows the structure of the dynamic phantom.

The phantom consists of 14.5 cm diameter, 1 cm thick acrylic disc. The diameter was chosen to simulate cardiac catheterization exams in clinical setting. In eight radiating directions, six 1 cm diameter holes were created in different depth representing targets. The positions of the targets were created in a concentric manner in 15 mm intervals with the distance from the center of the disc to the nearest target being 45 mm. The targets in the same direction have the same depths. The depths of the target in each direction measures 1.6 mm, 2.0 mm, 2.5 mm, 3.2 mm, 4.0 mm, 5.0 mm, 6.3 mm, and 8.0 mm. The different depths of the target holes generate different target contrast. Wires were placed to identify the holes closest and furthest to the center. The wires were fashioned in same lengths, diameter, and orientation so that the targets generated from them could also be evaluated.

When the height of the isocenter of the C‐arm from the floor is 105 cm and the distance between the focus and the FPD is 100 cm, the height of the table is between 85 to 105 cm from the floor, the 14.5‐cm radius of the dynamic phantom can be sufficiently imaged within 15.2 cm, 20.3 cm, and 25.4 cm fields of view. Figure 3(b) shows the function of the dynamic phantom. Reportedly, typical cardiac wall motion speed is assumed to be approximately 20 mm/s, based on a heart rate of 60 beats/min and 10 mm of movement of the cardiac wall between ventricular systole and diastole (20 mm combined).^(9)^ The coronary artery can be assumed to move in a similar fashion. To replicate this motion, a 3 rpm motor was used to rotate the dynamic phantom in this study. The phantom has variable speed ranging 1 rpm to 10 rpm. The cycle of rotation is shown in Fig. 3. The speed of the target motion was approximately 14.1 mm/s closest to the center of the phantom at 45 mm and increased incrementally toward the outer edge, with the maximum speed being approximately 37.7 mm/s at the outer most target. In terms of heart rate, approximately 14.1 mm/s would be equivalent to approximately 42 beats/min, whereas approximately 37.7 mm/s would be equivalent to 111 beats/min.

### D. Measurement of temporal changes in target pixel value

**Figure 4:**
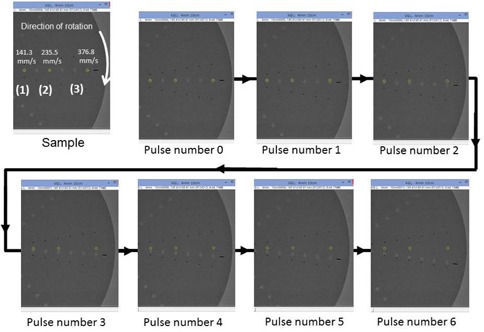
Setting of ROIs and method of measurement: (1): 141.3 mm/s; (2): 235.5 mm/s; (3): 376.8 mm/s.

As shown in Fig. 4, ROIs were set in 8 mm deep target and 300 fluoroscopic images were collected. Subsequently, seven continuous images were chosen for analysis. The images were chosen when the targets were aligning in the horizontal orientation (i.e., at 3 o'clock position) because of ease of reliably and setting up ROIs recurrently. The standard fluoroscopic image was designated “pulse number 0,” the subsequent image was designated “pulse number 1,” and the seventh image was designated “pulse number 6.” The average pixel value in circular ROIs with a 25 pixel diameter calculated using ImageJ (National Institutes of Health, Bethesda, MD) was used as the target pixel value. The ROIs were kept fixed on the image and the changes in pixel value were documented as the target passed through the ROIs over time by the rotation of the phantom. Due to the structure of the dynamic phantom, a part of the target on a ROI remains on a ROI during the next X‐ray exposure and the pixel value will contain residual value from prior exposure effectively creating instantaneous lag. Thus, the variation in the target pixel values as determined by calculating the area of the target that remains in the ROI (hereinafter, the “variation according to calculations”) was calculated at three speeds: 141.3 mm/s, 235.5 mm/s, and 376.8 mm/s. Next, changes in the targets were measured in collected fluoroscopic images at movement speeds of 141.3 mm/s, 235.5 mm/s, and 376.8 mm/s.

With the assistance of the manufacturer, three stages of intensity (intensity 1, 2, and 3) of forced image lag (hereinafter, “image lag intensity 1,” “image lag intensity 2,” and “image lag intensity 3”) were created in order to verify the ability of our phantom to detect a known instantaneous lag. Fluoroscopic X‐ray images were collected without forced image lag (hereinafter, “image lag intensity 0”) and with forced image lag. The four different intensities of forced image lag were then measured.

The measurements were done between 5 cm and 10 cm thickness (total of 10 cm and 20 cm thickness, respectively) acrylic boards to account for differences in body mass.

## III. RESULTS

### A. Relative pixel values in ROIs (Figs. 5 & 6)

**Figure 5:**
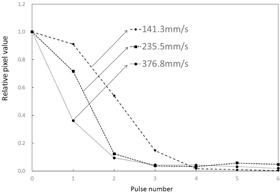
Changes in relative pixel values in ROI at different target movement speeds. Phantom thickness=10 cm.

Relative pixel values in ROIs by target speed are shown in Fig. 5. Figure 5 is a graph that shows the relative pixel value in the ROI at various target speeds with the measured value at the time of target overlap in the ROIs standardized to 1.0. Differences in relative pixel value in the ROI were observed at different target movement speeds, and when target movement was slower, the relative pixel value in the ROI was continuously higher. Furthermore, from pulse number 3 onward, the relative pixel value in the ROI infinitely approached 0, regardless of the speed of target movement.

**Figure 6:**
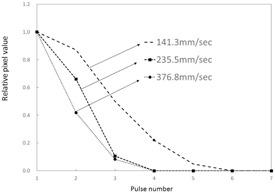
Changes in relative pixel values in ROI at different target movement speeds. Phantom thickness=20 cm.

There were no significant differences in temporal relative pixel value changes and in X‐ray fluoroscopic characteristics between the 10 cm and 20 cm acrylic board thickness groups.

### B. Variation in target change according to calculations

**Figure 7:**
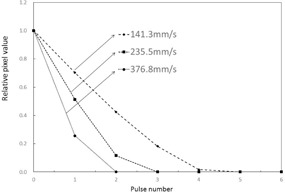
Variations in expected target pixel value changes derived from calculation.

The variation in target change according to calculations (Fig. 7), which was obtained from the dynamic specifications of the dynamic phantom, was similarly standardized to 1.0 when targets overlapped in the ROIs, and values were compared. Even when there was no forced lag, the measured relative pixel values in the ROI were higher than the variation in target change according to calculations.

### C. Intensity of forced image lag and temporal changes in target pixel values

As shown in 8, 9, measured relative pixel values in the ROI reflected changes in image lag intensity (between 1, 2, and 3) regardless of target movement speed. This is because measured pixel values in ROIs were higher than the variation in target according to calculations as the intensity of image lag increased from 1 to 2 to 3.

The instantaneous lag strengths and temporal signal changes in between 10 cm and 20 cm phantoms were identical. Nor were there any significant changes in the X‐ray spectral output changes.

**Figure 8:**
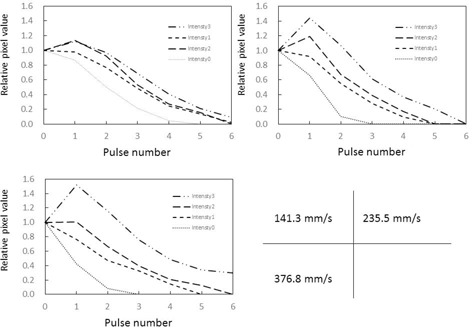
Temporal changes in relative pixel values in ROIs at different intensities of forced image lag. Phantom thickness=10 cm. Target movement speed compared between (1): 141.3 mm/s, (2): 235.5 mm/s, and (3): 376.8 mm/s.

**Figure 9:**
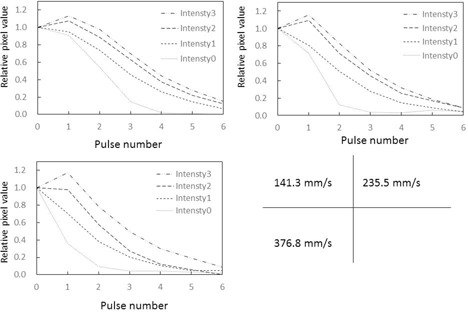
Temporal changes in relative pixel values in ROIs at different intensities of forced image lag. Phantom thickness=20 cm. Target movement speed compared between (1): 141.3 mm/s, (2): 235.5 mm/s, and (3): 376.8 mm/s.

## IV. DISCUSSION

### A. Relative pixel values in ROIs

Relative pixel values in ROIs are standardized to 1.0 when the target is aligned with the ROIs. When the relative pixel value in a ROI becomes “0” on the next pulse, no lag has occurred. As shown in Fig. 5, the relative pixel value in the ROIs infinitely approached 0 from pulse number 4 onward, due to target overlap and the disappearance of lag. A large relative pixel value in the ROI indicates that lag is occurring. However, besides lag, the relative pixel value in the ROIs includes target components that overlap with the ROIs. As can be seen in Fig. 5, the relative pixel values in ROIs tended to increase as target movement speed decreased, which is a characteristic of the dynamic phantom. This characteristic is thought to be due to overlap that arises from differences in movement speed of the targets positioned on the dynamic phantom and intensifies when the target is slower.

**Figure 10:**
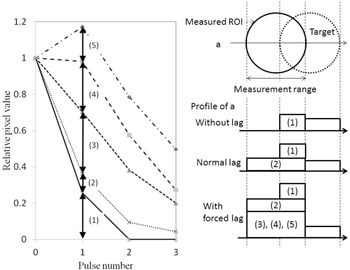
Association between intensity of forced image lag and relative pixel values in ROIs.

As shown in Fig. 10, forced image lag was induced and changes in lag intensity were accurately detected as changes in relative pixel value in the ROIs, thus verifying the ability of our phantom to detect know instantaneous lag. As the overlapping part of the target can be quantified, it is possible to calculate image lag from the relative pixel values in ROIs. Essentially, the difference between measured pixel values and pixel values according to calculations can be considered the lag component. Although the lag component consists of image lag and ghosting, this study only took measurements over periods of less than 1 s, so ghosting which take longer to be created should not contribute to lag. Therefore, the lag component calculated here can be implied to represent instantaneous lag.

The graphs in 11, 12 were created by subtracting calculated image lag values from the observed lag values in our phantom, and the corrected pixel values in the ROIs were charted. The effect of target movement speed on extraction of the image lag was evaluated. A target tends to linger longer when it moves more slowly and results in more lag. Conversely, fast‐moving targets exhibit a trend opposite to that of slow‐moving targets, where image lag decreases as the pulse number increases. For both slow and fast‐moving targets, an inverse phenomenon was seen in the relative pixel value of image lag as the pulse number increased. This is likely because the machine perceives the part of the target that overlaps on the ROIs during exposure to the next pulse as the target, so lag occurs at the next point of measurement. Essentially, it appears that lag builds up at lower target speeds. Pixel values observed from forced lag settings confirmed that our phantom is able to detect and quantify instantaneous lag. The dynamic phantom we developed in this study is effective for evaluating image lag in dynamic images. There were no differences in 10 cm and 20 cm thickness acrylic phantom thickness groups in lag strengths and temporal pixel value changes.

**Figure 11:**
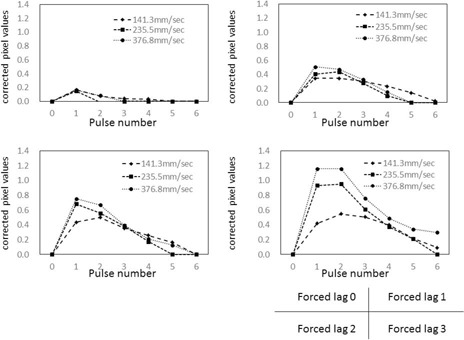
Relative pixel values in ROIs when measured values for image lag are subtracted from the variation according to calculations. Phantom thickness=10 cm. Comparison of signal movement speed expressed as only the difference from measured forced image lag.

**Figure 12:**
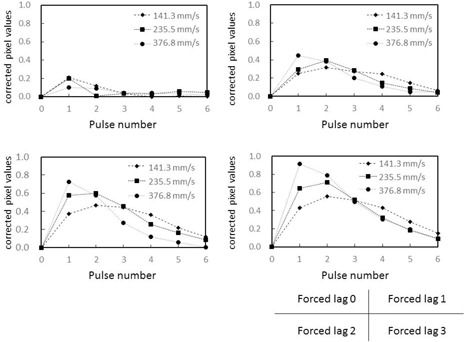
Relative pixel values in ROIs when measured values for image lag are subtracted from the variation according to calculations. Phantom thickness=20 cm. Comparison of signal movement speed expressed as only the difference from measured forced image lag.

### B. Structure of the phantom

Very few dynamic phantoms are commercially available. The most popular type of commercially available dynamic phantom has a highly concentrated area of target wires with different thicknesses. This is most likely to ensure optimum visualization of guidewires, which can cause severe vessel injury when mistreated. Visual evaluation is the main method used with present‐day commercially available dynamic phantoms. However, the wires used as targets in commercially available dynamic phantoms are too thin to be used for physical evaluation of image lag. Once clinical data are collected and standard values are determined, our dynamic phantom that can quantitatively evaluate image lag could be effective in routine checking as a tool for ensuring stable machine performance.

## V. CONCLUSIONS

We developed a dynamic phantom that can be used to physically evaluate image lag in dynamic images. This dynamic phantom has a unique measurement method. ROIs on the target are chosen and, with the position of ROIs kept fixed on the image, changes in pixel value are physically detected when the target passes through the ROIs over time and perceived as image lag. Thus, it was able to physically detect different intensities of image lag while maintaining the same intensity trends. Results suggest that our proposed checking technique using the dynamic phantom that we developed is useful for routine checking of FPD‐equipped cardiovascular machines, and could become an established method.

## ACKNOWLEDGMENTS

We sincerely thank all the radiological technologists at the Department of Radiological Technology of Showa University for their advice, guidance, and collaboration in this study.
